# Incorporation of Natural and Recombinant Collagen Proteins within Fmoc-Based Self-Assembling Peptide Hydrogels

**DOI:** 10.3390/gels8050254

**Published:** 2022-04-21

**Authors:** Mattia Vitale, Cosimo Ligorio, Ian P. Smith, Stephen M. Richardson, Judith A. Hoyland, Jordi Bella

**Affiliations:** Division of Cell Matrix Biology & Regenerative Medicine, School of Biological Sciences, Faculty of Biology, Medicine and Health, Manchester Academic Health Sciences Centre, The University of Manchester, Manchester M13 9PT, UK; mattia.vitale@manchester.ac.uk (M.V.); cosimo.ligorio@manchester.ac.uk (C.L.); ian.smith-9@postgrad.manchester.ac.uk (I.P.S.); s.richardson@manchester.ac.uk (S.M.R.); judith.a.hoyland@manchester.ac.uk (J.A.H.)

**Keywords:** peptide hydrogel, recombinant collagen, diffusion protocol, fibrosarcoma cell, integrin

## Abstract

Hydrogel biomaterials mimic the natural extracellular matrix through their nanofibrous ultrastructure and composition and provide an appropriate environment for cell–matrix and cell–cell interactions within their polymeric network. Hydrogels can be modified with different proteins, cytokines, or cell-adhesion motifs to control cell behavior and cell differentiation. Collagens are desirable and versatile proteins for hydrogel modification due to their abundance in the vertebrate extracellular matrix and their interactions with cell-surface receptors. Here, we report a quick, inexpensive and effective protocol for incorporation of natural, synthetic and recombinant collagens into Fmoc-based self-assembling peptide hydrogels. The hydrogels are modified through a diffusion protocol in which collagen molecules of different molecular sizes are successfully incorporated and retained over time. Characterization studies show that these collagens interact with the hydrogel fibers without affecting the overall mechanical properties of the composite hydrogels. Furthermore, the collagen molecules incorporated into the hydrogels are still biologically active and provide sites for adhesion and spreading of human fibrosarcoma cells through interaction with the α2β1 integrin. Our protocol can be used to incorporate different types of collagen molecules into peptide-based hydrogels without any prior chemical modification. These modified hydrogels could be used in studies where collagen-based substrates are required to differentiate and control the cell behavior. Our protocol can be easily adapted to the incorporation of other bioactive proteins and peptides into peptide-based hydrogels to modulate their characteristics and their interaction with different cell types.

## 1. Introduction

Hydrogels are three-dimensional (3D) networks made of hydrophilic polymers that can entrap and hold water while forming self-supporting systems through chemical or physical cross-linking of individual polymer chains [1]. Importantly, they can serve as scaffolds for tissue engineering applications due to their structural and functional similarity with the extracellular matrix (ECM). In fact, natural ECM is a complex meshwork consisting of macromolecules and in some tissues minerals where resident cells interact with their surroundings in a 3D network [2]. One of the challenges when developing new hydrogel-based biomaterials is to design an ECM-mimicking nanofibrous network while keeping the system simple, biocompatible and able to modulate cellular behaviour with minimal chemical modification. Peptide-based hydrogels that self-assemble into 3D networks represent a valid approach to this problem [3]. However, the hydrogelator peptides themselves may be non-interactive to cells or provide low cell adhesion. Therefore, biologically active components can be attached by chemical modification of these peptides without interfering with the self-assembly mechanism. The biologically active groups are then exposed on the surface of the hydrogels and hence are available for interaction with cells [4,5]. It has been shown that incorporation of small functional groups such as amines and phosphates can induce stem cell differentiation towards bone and adipose cells [6], whereas addition of integrin-binding motifs, such as RGD, can improve cell adhesion and viability [7,8]. Similarly, hydrogels can be modified with inorganic components, used as “nanofillers”, to form peptide-nanocomposite hydrogels with enhanced mechanical properties and improved cell differentiation activity [9,10,11,12,13,14,15].

Hydrogel modification through incorporation of collagen molecules has gained widespread popularity in tissue engineering applications. Collagen is the most abundant protein in the body, providing strength and structural stability to tissues as well as cell-binding motifs [16,17]. Moreover, its abundance in the ECM makes it an ideal candidate for hydrogel modifications. For instance, it has been shown that hydrogels modified with collagen peptides showed enhanced cell-adhesion properties and control of stem cell differentiation [18,19]. Indeed, collagen modification of hydrogels can be challenging due to heterogeneity of fabrication protocols used by different research groups and, most importantly, potential undesirable properties of the collagen source [20]. Animal-derived collagen, for example, is most commonly harvested from bovine and porcine sources, which present significant risks of zoonotic disease transmission and immunogenicity [21]. Extraction of collagen from animal connective tissues is also hampered by the large proportion of material that is crosslinked and insoluble [22]. Thus, animal-derived collagens often require use of enzymes such as pepsin, which may digest the collagen proteins to some extent and decrease the mechanical properties of the final biomaterial. These inherent limitations have fuelled the exploration of alternative sources to animal-derived collagen such as the chemical synthesis of collagen-like peptides or the production of collagens or collagen-like proteins using recombinant techniques [23,24]. In fact, the production of collagen via recombinant technology represents a valuable, cost-effective and most importantly, safe alternative for collagen production [25].

In this work we used a minimalistic approach to incorporate natural and recombinant collagen proteins within 9-fluorenylmethoxycarbonyl (Fmoc)-based self-assembling peptide hydrogels (SAPHs). It has been shown that short Fmoc-protected peptides are effective low molecular weight gelators that form rigid nanotube structures that lead to hydrogel formation without further need of crosslinking agents. Furthermore, by tailoring the amount of peptide powder, the hydrogel’s resulting stiffness can be modulated accordingly [26,27,28]. This makes Fmoc-based hydrogels potentially attractive for different tissue engineering applications. Here, we used Fmoc-diphenylalanine/Fmoc-serine (Fmoc-FF/S) peptide hydrogels to develop new customised peptide/collagen composite hydrogels. We used our prior expertise on the successful incorporation of inorganic crystalline materials into Fmoc-based hydrogels [13] to develop a quick and effective way of modifying these hydrogels with natural, synthetic or recombinant peptides/proteins of a wide range of molecular weights and sizes. We demonstrate here effective incorporation of collagen proteins via diffusion within peptide hydrogels and report the mechanical characterization of collagen-modified hydrogels via rheology and their biological characterisation through cytocompatibility studies. Our data demonstrate that our protocol for protein incorporation produces hybrid peptide/collagen hydrogels where the added proteins are well integrated and retained within the host peptide-based scaffolds without the need of chemical modification. Furthermore, incorporated collagens do not interfere with the self-assembly mechanism of the hydrogels or with the mechanical properties of the resulting biomaterial but serve to provide extra integrin-binding sites that improve cell adhesion and spreading. Furthermore, we believe that this protocol can be adapted to the incorporation of both small and large proteins within Fmoc-based peptide hydrogels providing a simple, yet effective way of creating peptide/protein composite hydrogels to be used in tissue engineering and regeneration applications.

## 2. Results and Discussion

### 2.1. Characterization of the Proteins Used in Hydrogel Modification

**Figure 1:**
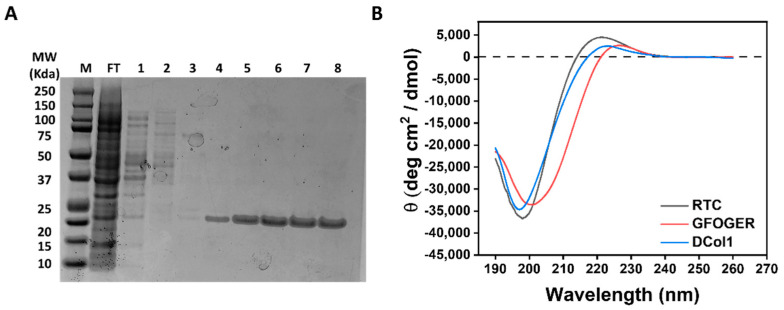
Protein characterization. (**A**) SDS-PAGE analysis of the purification of DCol1 by nickel-affinity chromatography. Lanes: M, molecular weight markers; FT, flow through; 1–2, wash fractions with 60 mM imidazole; 3–4, elution fractions with 250 mM imidazole; 5–8 elution fractions with 1 M imidazole. (**B**) CD spectra at 4 °C of RTC (grey), DCol1 (blue) and GFOGER peptide (red). The vertical axis measures mean residue ellipticity θ in degrees cm^2^ dmol^−1^. CD data were collected between 190 and 260 nm.

Three different collagen molecules were chosen for incorporation within the self-assembling peptide hydrogels (Table 1): Rat Tail Collagen (RTC); a 42-amino acid collagen peptide containing a GFOGER integrin-binding motif (GFOGER peptide); and an engineered recombinant collagen-like mini protein produced in house (DCol1). In addition, recombinant enhanced green fluorescent protein (eGFP) was used as a fluorescent reporter to follow the protein incorporation and retention within the hydrogel during optimization of the modification protocol [29]. RTC has been extensively used as a positive control to study cell–matrix interaction within 3D hydrogels [30,31], and it has been chosen as the largest protein (approximately 300 kDa molecular weight for the full-length trimer) to be incorporated within our system. The GFOGER peptide is the shortest version of collagen used here (11.24 kDa molecular weight for the trimer), containing a GFOGER motif (O is 4-hydroxyproline) that binds integrins α1β1 and α2β1 [32]. DCol1 is a designed recombinant collagen-like protein containing a GFPGER motif which is also able to bind α1β1 and α2β1 integrins. Proteins were characterized by SDS-PAGE and circular dichroism (CD) (Figure 1). Purification of DCol1 via nickel-affinity chromatography showed high production and purification yields (Figure 1A). The triple-helical conformation of the three collagen molecules was studied by CD spectroscopy. The CD spectra of RTC, GFOGER and DCol1 at 4 °C in CD buffer (Figure 1B) showed the characteristic features of triple helical collagen: a band of positive ellipticity (around +3000 to +5000 deg cm^2^ dmol^−1^) with a maximum at around 220 nm and a deep band of negative ellipticity (around −35,000 deg cm^2^ dmol^−1^) with a minimum around 198 nm [33,34,35]. Both these features are associated with the polyproline II conformation [36], and they are characteristic of the collagen triple helix.

### 2.2. Protein Incorporation into Self-Assembly Peptide Hydrogels

**Figure 2:**
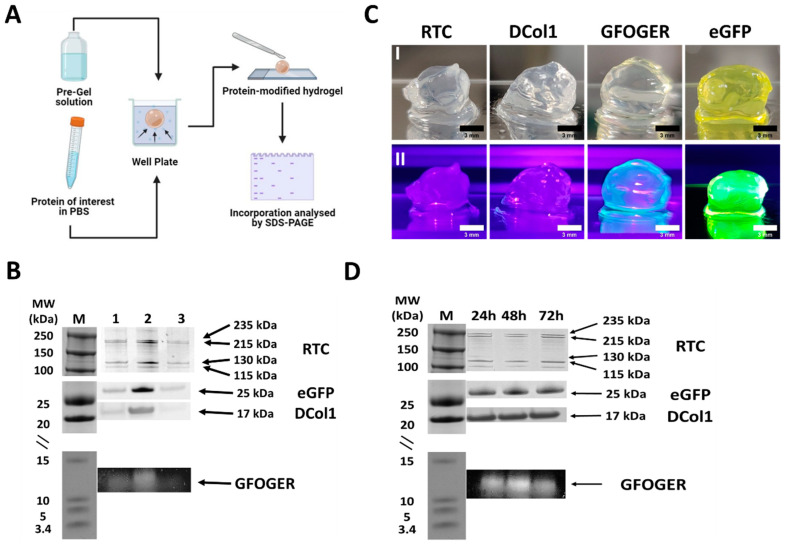
Hydrogel modification. (**A**) Schematic representation of the optimised protocol used to incorporate proteins within hydrogels. (**B**) SDS-PAGE analysis of the proteins incorporated into the Fmoc-FF/S hydrogels. Lanes: M, molecular weight markers; 1, protein stock solution; 2, protein incorporated into the hydrogel; 3, remaining protein into the well. Arrows (right) indicates protein bands at their corresponding molecular weights. (**C**) Photograph of spheroid-like hydrogels incorporating proteins under visible (**I**) and UV (**II**) light. (**D**) SDS-PAGE analysis of protein retention inside the hydrogels over time. Lanes: M, molecular weight markers; 1, 2, 3 show protein retention after 24, 48, 72 h, respectively. Arrows (right) indicates protein bands at their corresponding molecular weights.

Proteins with a wide range of molecular weights and sizes were successfully incorporated into Fmoc-FF/S hydrogels by simple diffusion, without need for any chemical modifications (Figure 2A), as demonstrated by SDS-PAGE analysis (Figure 2B) (full SDS-PAGE blot is available in Figure S1). Protein incorporation was comparable to the control bands (Figure 2B). We believe that, although samples obtained from hydrogel sections (Figure 2B, lane 2) showed a darker band than the controls (Figure 2B, lane 1), the stronger intensity of the band is caused by the hydrogels acting as a “concentrator” of protein in a constrained space. Indeed, the protein in the stock solution passed from a liquid form to being concentrated within a spheroid due to a volume shrinkage. A similar phenomenon was also shown by Kim and co-workers as they demonstrated that the fluorescence intensity of graphene oxide was much greater in a limited space as the cell pellet [37]. Importantly, hydrogels incorporating the collagen protein showed a significantly stronger band than the remaining protein solutions in the well (Figure 2B, lane 3), suggesting a clear protein uptake. The resulting spheroids were stable and formed a self-supporting hydrogel structure, as shown in Figure 2C. Interestingly, hydrogels incorporating eGFP and fluorescently labelled GFOGER showed a bright, green color when exposed to UV light (Figure 2C(II)) indicating that the proteins were diffused throughout the whole scaffold without affecting their chromophore fluorescence. Finally, we analyzed the protein retention over time. As shown in Figure 2D, SDS-PAGE showed consistent dark bands for all of the tested constructs, suggesting that all the proteins were successfully retained for at least the first 72 h. Different co-assembled peptide hydrogels systems of using alternative mechanisms have been explored [5,38]. In particular Stupp and co-workers have described a similar phenomenon when self-assembling peptide amphiphiles are mixed with high molecular weight polysaccharide hyaluronic acid. Liquid–liquid interaction results in the formation of hierarchically orientated sacs and membranes due a mixture of both osmotic pressure of ions and strong electrostatic interactions [38]. However, in this case, electrostatic interactions are not expected to play a significant role in the incorporation of the proteins into the Fmoc-FF/S peptide hydrogels, at least for the three collagen molecules (see Table 1 for their predicted values of pI). Thus, we believe that the incorporation of collagen proteins within our system is mostly driven by diffusion of the proteins into the hydrogel mesh, with the two components (i.e., Fmoc-FF/S and collagens) co-assembling upon contact.

**Figure 3:**
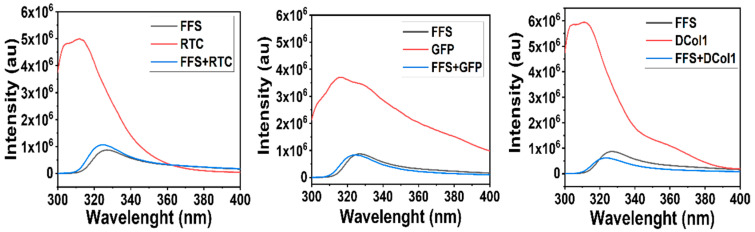
Fluorescence spectroscopy spectra of Fmoc-FF/S peptide (grey), collagen (red) and Fmoc-FF/S-collagen solutions (blue) prepared in PBS (pH = 7.4). Excitation wavelength 280 nm; emission wavelength recorded within the 300–450 nm range.

Moreover, interactions of a hydrophobic nature may also be involved when forming the hydrogels. As shown by the fluorescence spectroscopy analysis, each protein shows a fluorescence emission peak in the 320–380 nm region, where the fluorescence of tyrosine and tryptophan side groups is observed (Figure 3) [39]. The intensity of this peak is significantly lower when the proteins are mixed 1:1 *v*/*v* with the Fmoc-FF/S pre-gel solution. The dramatic reduction of fluorescence intensity reveals the existence of a strong interaction between the fluorescent groups in these collagen proteins and the Fmoc-FF/S pre-gel solution. We believe that as the self-assembly mechanism occurs, the hydrogels act as a “sink”, where the proteins passively diffuse through the hydrogels’ mesh until an equilibrium is reached after 24 h (Figure 2B) as previously demonstrated by Sassi et al. [40].

### 2.3. Hydrogels Microstrusture

**Figure 4:**
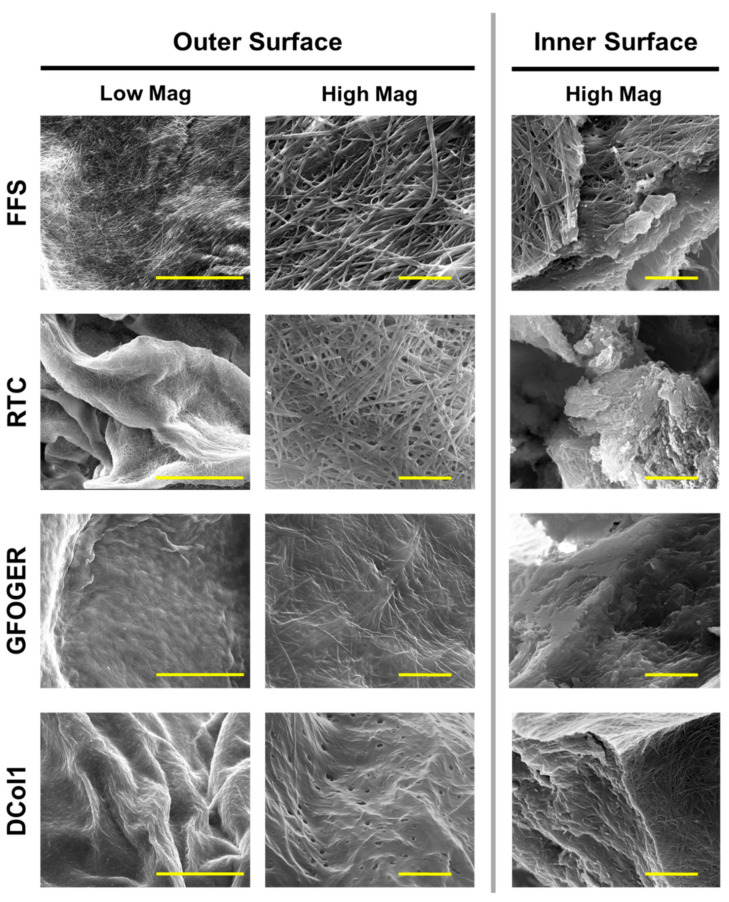
Scanning electron microscopy images showing the outer (**left**) surface morphology of Fmoc-FF/S and the collagen-modified hydrogels and a cross section showing the inner (**right**) surface morphology at lower and higher magnification, respectively. Low magnification scale bar is 50 µm; high magnification scale bar is 5 µm.

Hydrogel microstructures were also assessed using scanning electron microscopy (SEM). In addition to imaging the outer hydrogel surface, the gels were sliced to expose and allow imaging of the inner surface (Figure 4). Unmodified Fmoc-FF/S hydrogel shows the typical nanofibrous structure consisting of bundles of supramolecular stacks [27]. Additionally, by exposing its inner surface, we observed concentric lamellae as typically shown for the Fmoc-FF/S system where diffusion is involved [41]. The incorporation of different collagen proteins changed the hydrogels’ surface features and morphologies according to protein size. The incorporation of the biggest protein, RTC, showed differences with Fmoc-FF/S only on the inner surface of the scaffold where this appeared to be much more disorganised than the rest of the hydrogel. In contrast, the outer surface of the Fmoc-FF/S/RTC showed morphological similarity with the microstructures observed on the outer surface of Fmoc-FF/S. We speculate that smaller collagen peptides such as GFOGER and DCol1 may diffuse more efficiently into the co-assembled systems, as the final structures of the hybrid hydrogels appeared more compact and less disorganised. For this reason, we envisage that the size and shape of the diffusing proteins will dictate a more or less efficient incorporation.

### 2.4. Mechanical Characterization of the Hydrogels

**Figure 5:**
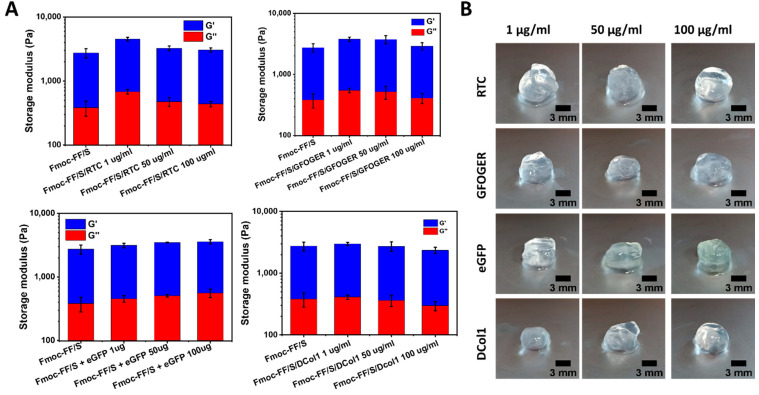
Analysis of the mechanical properties of the hydrogels studied. (**A**) Storage and loss moduli (0.02% strain, 1 Hz) of Fmoc-FF/S hydrogels with and without collagen at different concentrations (1, 50, 100 µg/mL). (**B**) Photographs of spheroids of Fmoc-FF/S with different collagen construct at different concentrations.

In order to assess the effect of protein incorporation on the final hydrogels’ mechanical properties, we analyzed the viscoelastic properties of the protein-modified hydrogels via rheology using unmodified Fmoc-FF/S hydrogel as control. Fmoc-FF/S with and without RTC, GFOGER, eGFP and DCol1 at different concentrations (1, 50 and 100 μg/mL) were subjected to an amplitude sweep experiment (strain range 0 to 100%, frequency 1 Hz, gap size 500 μm) at 37 °C. As can be seen from Figure 5A, no significant difference in the storage modulus G′ (used here as a measure of stiffness) was reported when hybrid hydrogels were formed. Moreover, the G′ was consistently higher than the loss modulus (G″) for all the formulation tested (Figure S2). This indicates that Fmoc-FF/S was able to incorporate all the proteins of interest while still self-assembling and forming self-supporting spheroid shaped hydrogels (Figure 5B). The incorporation of the different proteins did not affect the “bulk” properties of the final peptide/protein hybrid hydrogels, as the stiffness values showed neither a positive nor a detrimental effect to the final mechanical properties, compared to the unmodified Fmoc-FF/S peptide hydrogel.

### 2.5. Collagen-Modified Hydrogels as Platform for Cell Culture

**Figure 6:**
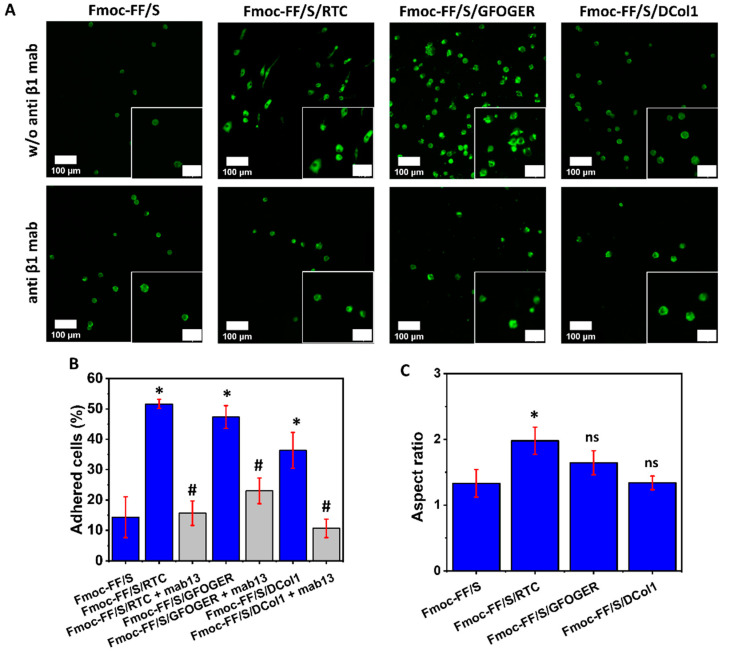
HT1080 cell adhesion and cell spreading. (**A**) F-actin staining of HT1080 cells after 24 h culture on the different collagen hydrogels (green: F-actin, Alexa Fluor 488 phalloidin; scale bars 100 µm, inset 50 µm) with and without pre-incubation with mAb13 (anti β1) antibody. (**B**) Analysis of cell adhesion on the different collagen-modified hydrogels without (blue bars) and with (grey bars) pre-incubation with mAb13 antibody. (**C**) Spread HT1080 cells show a mean aspect ratio above 1 while rounded shaped cells are reflected by an aspect ratio 1. Data shown as mean ± SD, *n* = 43, */# *p* < 0.05, ns (not significant). Significance for each group is relative to Fmoc-FF/S (*), with the exception of groups shown in the grey columns which are in comparison to their respective blue columns (#).

To assess the biological activity of the collagen-modified hydrogels, HT1080 cells were used. They are a human fibrosarcoma cell line that is often used for collagen adhesion experiments, and they are known to express high levels of α2β1 integrin on their surface, which is a major cellular receptor for collagen [42,43]. Hydrogels modified with RTC, GFOGER peptide and DCol1 were tested. eGFP was not used for this experiment as it does not provide any cell binding site [44]. Two major parameters were evaluated to assess the properties of the hybrid hydrogels: cell adhesion and cell spreading. One representative protein concentration (100 μg/mL) was used throughout this experiment. Compared to the unmodified Fmoc-FF/S hydrogels, hybrid hydrogels incorporating collagen provided a significantly higher cell adhesion, as can be seen from Figure 6A. As expected, RTC-modified hydrogel provided the maximum cell adhesion (51 ± 1% vs. 14 ± 6.7%, *p* < 0.05). Similarly, Fmoc-FF/S + GFOGER provided greater cell attachment than unmodified peptide systems (47 ± 3.7% vs. 14 ± 6.7%, *p* < 0.05). Among all the collagen mimics incorporated, DCol1, provided the lowest cell adhesion properties (36 ± 5.9%), although still significantly higher than the unmodified Fmoc-FF/S hydrogel (14 ± 6.7%, *p* < 0.05) (Figure 6B, blue bars). We believe that two main factors may be affecting this: firstly, the effect is measured after 24 h, and it could be mitigated due the physiological adaptation of the cells into a new microenvironment [45]; secondly, we hypothesize that, due to small size of DCol1 (17 kDa), easy access to integrin binding sites could be hindered within the hydrogel mesh.

Secondly, we analyzed the capability of HT1080 cells to spread on the modified hydrogels. Analysis of the aspect-ratio (Figure 6C) showed that RTC and GFOGER-modified hydrogels were able to support cell-spreading, as also shown in the upper panel of Figure 6A. However, HT1080 cells showed no spreading and a rounded morphology when they were cultured on the DCol1-modified hydrogels. This effect was already seen in previous experiments on TCP (data not shown) and the reasons for this are still unknown and will require further investigation. We hypothesize that it could be due to an intrinsic toxic component within the DCol1 structure, whose effect becomes more pronounced when the protein is more concentrated [46]. This aspect is currently under investigation and would be the subject of a future work. Finally, to determine whether the cells were directly binding to the collagen decorated on the hydrogel via their β1-integrin, a blocking antibody was used. Optimal concentration of anti-β1 antibody was previously optimized as illustrated in Figure S3. As can be seen from the lower panels in Figure 6A, when cells were pre-incubated with mAb13 antibody, they showed less or no cell spreading, maintaining a well-rounded morphology compared to the Fmoc-FF/S hydrogel. Additionally, cell adhesion was significantly lower than that of the untreated cells. In fact, shown in Figure 6B (grey bars), a significant reduction of ~30% of cell adhesion was observed for RTC-modified hydrogel (51 ± 1% vs. 15 ± 4% *p* < 0.05) as well as a ~50% decrease for Fmoc-FF/S/GFOGER hydrogels (47 ± 3.7% vs. 23 ± 4.2%, *p* < 0.05) compared to the untreated cells. Similarly, DCol1-modified hydrogels showed a ~27% decrease in cell adhesion (36 ± 5.9% vs. 10 ± 3%, *p* < 0.05). Nevertheless, they did not show any significant difference with the unmodified Fmoc-FF/S peptide hydrogel. It has been demonstrated that the integrin α2β1 is one of the major receptors for collagens protein [47,48]. Our findings are consistent with these studies suggesting that the β1-integrin subunit acted as a mediator, linking cells to the collagen modified hydrogels for cell-biomaterial interactions.

## 3. Conclusions

In this study, we have demonstrated a new and efficient diffusion-based approach to incorporate natural, synthetic and recombinant collagen proteins within Fmoc-based self-assembling peptide hydrogels. Our protocol of incorporation is effective in creating new collagen-containing hydrogels without the need for a prior chemical modification. We believe that a passive diffusion occurs when the collagen protein of interest makes contact with the pre-gel peptide solution creating a co-assembled system, with the protein becoming entrapped in the hydrogel nanofibrillar mesh as the self-assembly mechanism occurs. Moreover, hydrophobic interactions may be involved between the two counterparts, stabilizing the overall networks of peptide/collagen hydrogels. Furthermore, the size of the incorporated collagen impacts the nanostructure of the composite hydrogel. As shown by our EM images, the larger molecules of type I collagen generate more disorganized co-assembled systems than the smaller recombinant collagen or synthetic peptide. Nevertheless, our material characterization indicates that the incorporation of collagen does not affect the mechanical properties of the resulting hydrogels at any chosen concentration (the difference in G′ is not significant). We consider this a positive result as we did not want to alter the original stiffness of the hydrogels, as such alteration could have had consequences for cellular mechanotransduction signaling and cell behavior. [49,50].

Furthermore, the addition of collagen to the Fmoc-FF/S introduces additional biological features that are missing in the unmodified hydrogels. Firstly, our collagen-modified hydrogels show enhanced cell adhesion with a significantly higher number of adhered cells compared to the unmodified hydrogels. This confirms that the protocol developed here is able to successfully incorporate collagen protein molecules that remain biologically active. Secondly, HT1080 cells appear to spread on the collagen-modified hydrogels, apart from Fmoc-FF/S/DCol1. This is something already expected and in line with our preliminary data on 2D plastic. Finally, we have demonstrated that the cell adhesion is mediated via the β1-integrin subunit as mab13 pre-treated cells lose their ability to attach to the hydrogels and do not spread. Numerous methods can be used to modify hydrogels in order to incorporate bioactive adhesion molecules [51]. We believe that our protocol of incorporation can be exploited to incorporate collagen-like proteins, as well as other functional peptides containing the RGD cell-binding motif, and bioactive components such as anabolic growth factors, without modifying the chemical structure of the hydrogels and without using toxic cross linkers. As such, we have demonstrated a cost-effective way of creating peptide/protein co-assembled, composite hydrogels for use in tissue engineering and cell culture applications.

## 4. Materials and Methods

### 4.1. Materials

Table 1 summarises the different peptides and proteins used in this work for hydrogel preparation and modification. Fmoc-FF/S (a mixture of Fmoc-diphenylalanine and Fmoc-serine peptides, 1:1 ratio) was obtained from Biogelx Ltd., Motherwell, UK (batch number FFS052RM). Peptide quality (97% purity) was assessed at Biogelx via High Performance Liquid Chromatography (HPLC). Rat Tail Collagen (RTC, C3867, Sigma-Aldrich, Welwyn Garden City, UK) was obtained as an aqueous solution in 20 mM acetic acid, at a stock concentration of 3 mg/mL and 95% purity. The 42-amino acid integrin-binding GFOGER peptide [52] (Table 1) was obtained from Cambcol Laboratories Ltd., Cambridge, UK as lyophilized powder and dissolved to a final concentration of 1 mg/mL in Dulbecco’s Phosphate Buffered Saline (PBS, Sigma-Aldrich). For simplicity we will include the GFOGER peptide in the general category of “proteins” used here for hydrogel modification. Recombinant eGFP (Table 1) was already available in purified form, previously produced from an in house pET15b-eGFP expression vector [53].

### 4.2. Recombinant Collagen Design and Purification

A 165-amino acid recombinant collagen was designed in house by fusing a short collagen sequence (72 amino acids) with a trimerization domain from a collagen-like protein from *E. coli* [46]. The amino acid sequence of designed collagen DCol1 is shown in Table 1. Gene synthesis, subcloning and expression tests with different *E. coli* strains was carried out by ProteoGenix, Schiltigheim, France [54]. Best expression conditions were obtained with the protein expression vector pET28b using T7 Express cells. Bacterial pellets containing expressed DCol1 were re-suspended in 20 mL lysis buffer (PBS, lysozyme, 5 mM Imidazole, pH 7.5) with a protease inhibitor tablet (cOmplete Mini EDTA-free protease inhibitor, Roche, Basel, Switzerland). Cells were homogenized using a French cell press (Thermo IEC, FA-078A, Waltham, MA, USA) with a miniature pressure cell (FA-003) working at 20,000 psi. Disrupted cells were collected on ice before centrifugation for 2 h at 12,500 rpm and 4 °C. The pellets of cell debris were discarded, and the supernatant containing soluble protein was mixed with 2 mL of Nickel-nitrilotriacetic resin (HisPur™ Ni-NTA Thermo Scientific, Waltham, MA, USA) previously equilibrated with 10 mL binding buffer (PBS, 5 mM Imidazole, pH 7.5). The resin-protein suspension was incubated on a roller unit overnight at 4 °C, with continuous mixing to maximise binding. The following day gravity columns were prepared with the resin-protein suspension. The unbound fraction was collected. The column was then washed twice with 20 mL of washing buffer (PBS, 60 mM Imidazole, pH 7.5) to remove contaminants. The column was further washed with 250 mM Imidazole in PBS and the protein finally was eluted using 1 M Imidazole in PBS. All fractions were analysed by SDS-PAGE to determine which of them contained the purified protein. Dialysis tubing (Biodesign™ D100, 8000 MWCO, Thermo Scientific, Waltham, MA, USA) was used to remove the unwanted imidazole from the desired fractions by dialysing them against fresh PBS.

### 4.3. SDS-PAGE

Pre-cast NuPAGE™ 4–12% Bis-Tris mini protein gels (ThermoFisher Scientific), 1.0 mm gel thickness and 10 wells, were used with Invitrogen™ mini gel tanks. Samples were prepared by diluting 15 μL of analyte in 10 μL NuPAGE™ 4X LDS loading buffer (ThermoFisher Scientific) before heating at 95 °C for 5 min on a heating block (HB120-S, Scilogex, Rocky Hill, CT, USA). Hydrogels samples were mixed with loading buffer up to a final volume of 100 μL to help dissolving the gel. After heating, 10 μL of each sample was loaded alongside 5 μL of prestained protein ladder, 10 to 250 kDa (PageRuler™ Plus, ThermoFisher Scientific)**.** All gels were run for 1 h at a constant voltage (120 V) using NuPAGE™ MES SDS running buffer (ThermoFisher Scientific). Gels were stained overnight using Coomassie blue (InstantBlue™, Expedeon, Heidelberg, Germany) and imaged using a compact scanner (CanoScan LiDE 220, Tokyo, Japan).

### 4.4. Circular Dichroism Spectroscopy

Secondary structures of the RTC and DCol1 proteins and the GFOGER peptide were analysed by circular dichroism (CD) spectroscopy using a Jasco^®^ J-810 spectropolarimeter equipped with a Peltier temperature controller. Samples were diluted to a concentration of ~0.5 mg/mL in CD phosphate buffer (10 mM K_2_HPO_4_, 10 mM KH_2_PO_4_, 150 mM KF, pH 7.4) [55]. CD spectra were measured between 190 nm and 260 nm at 4 °C using a 1 mm pathlength CD-matched quartz cuvette (Starna Scientific, Ilford, UK). Data were collected every 0.2 nm with a 1 nm bandwidth. Spectral baselines were corrected by subtracting the spectrum of CD phosphate buffer (blank) collected under the same conditions.

### 4.5. Hydrogel Modification Protocol

Fmoc-FF/S peptide powder (13 mg) was dissolved in 1 mL of sterile deionized H_2_O to a concentration of 15 mM, hereafter referred as pre-gel solution. Collagen proteins were incorporated within the hydrogels by diffusion. In order to follow the incorporation of GFOGER, the peptide was prior labelled with 10 µM NHS-Fluorescein (Sigma-Aldrich) for 30 min at room temperature. Briefly, the proteins of interest were diluted up to the desired concentration in PBS; 1.5 mL of this solution was then placed into a 24-well plate (Corning, New York, NY, USA). A 1.5 mL solution of PBS was used as a protein-free control well. Approximately 300 µL of pre-gel solution were pipetted into the same well forming a spheroid-shape hydrogel which encapsulated the protein as the crosslinking process took place. After 24 h, the resulting spheroids were scooped out of the well, and protein incorporation/retention was analysed by cutting a slice of each hydrogel and by loading them into an SDS-PAGE as described in Section 4.3.

### 4.6. Fluorescence Spectroscopy

Fluorescence spectroscopy measurements were performed on RTC, eGFP and DCol1 on PBS (100 µg/mL, pH 7.4), on Fmoc-FF/S pre-gel solutions (15 mM, 13.2 mg/mL) and on 1:1 *v*/*v* mixtures of each protein in PBS and Fmoc-FF/S pre-gel solutions. Measures were carried out at room temperature using a FluoroMax-4 spectrofluorometer (HORIBA, Northampton, UK). Samples were loaded into 0.2 cm path length quartz cuvettes. Fluorescence spectra were acquired using a 280 nm excitation wavelength and emission recorded in the 300–450 nm range.

### 4.7. Scanning Electron Microscopy (SEM)

The morphologies of Fmoc-FF/S hydrogels with and without incorporated collagens were analysed by Scanning Electron Microscopy (SEM, Thermo Fisher Scientific, Loughborough, UK). Briefly, hydrogels were prepared by pipetting ~300 μL of the pre-gel solutions into Thin-Cert well inserts (0.4 μm pore size Greiner Bio-One Ltd., Stonehouse, UK). The inserts were then placed into 24-well plates and incubated at 37 °C with a total volume of 1.3 mL PBS containing the protein of interest to fully crosslink the hydrogels. After 24 h, hydrogels were fixed in 2.5% (*w*/*v*) glutaraldehyde (Sigma-Aldrich, Welwyn Garden City, UK) and 4% (*w*/*v*) paraformaldehyde (Sigma-Aldrich, Welwyn Garden City, UK) in 0.1 M HEPES buffer (Sigma-Aldrich, Welwyn Garden City, UK). After rinsing the samples in PBS, all samples were dehydrated in a graded ethanol (EtOH) series (25, 50, 75, 95, and 100% *v*/*v* EtOH/water). Samples were maintained at 100% EtOH and dried in a K850 Critical Point Drier (CPD, Quorum Technologies, Lewes, UK). After the CPD step, samples were transferred into metallic pins and coated with gold palladium alloy using an SC7620 Mini Sputter Coater (Quorum, Lewes, UK). Samples were then imaged on a Quanta 250 FEG SEM (Thermo Fisher Scientific, Loughborough, UK) at 20 kV.

### 4.8. Mechanical Properties of the Hydrogels

The rheological properties of Fmoc-FF/S hydrogels with and without incorporated proteins (RTC, GFOGER, DCol1 or eGFP) were analysed via a rheological amplitude sweep test on a Discovery HR-2 rheometer (TA instruments, New Castle, DE, USA). Each hydrogel sample was tested in the 0–100% shear strain range with a frequency of 1 Hz, gap size of 500 μm, temperature of 37 °C, and rheometer’s plate diameter of 20 mm. The rheometer upper head was lowered to the desired gap size and a soak time of 180 sec was used for equilibration. A solvent trap was employed to minimise sample evaporation. Once the rheological spectra were collected, representative storage and loss moduli at 0.02% and frequency of 1 Hz were selected for the summary rheology plots.

### 4.9. Hydrogel Cell Adhesion and Spreading

Human Fibrosarcoma HT1080 cells (ATCC CCL-121) were maintained in tissue culture polystyrene (TCPS) using Dulbecco’s Modified Eagle Medium (DMEM, Gibco, Loughborough, UK) containing 10% (*v*/*v*) foetal bovine serum (FBS) and 5% (*v*/*v*) Penicillin-Streptomycin-Amphotericin antibiotic mixture (PSA, 100 units/mL penicillin, 100 μg/mL streptomycin, 0.25 μg/mL amphotericin) (Sigma-Aldrich, Welwyn Garden City, UK). When reaching confluency, cells were gently detached from the tissue culture flask by adding 4 mL of Trypsin-EDTA (Sigma-Aldrich Welwyn Garden City, UK) and pelleted by centrifugation (400× *g* for 5 min). After cell counting, fresh culture medium was added to obtain the desired cell density. Collagen-modified hydrogels were prepared 24 h in advance as described in Section 4.5. However, when used for cell culture, ~250 μL of pre-gel solution was pipetted into the inner well of a 35 mm glass bottom dish for confocal microscopy (VWR, Leicestershire, UK, 734–2905), followed by the addition of 2 mL of the protein of interest in PBS to allow crosslinking and collagen incorporation. Cell adhesion and cell spreading analysis were evaluated as described by Humphries et al. [56]. Briefly, non-specific bindings were blocked by adding 1 mL of heat-denatured high grade Bovine Serum Albumin (BSA, Sigma-Aldrich, Welwyn Garden City, UK), at 10 mg/mL concentration for 1 h. Then, 2 mL of cell suspension (4 × 10^5^ cell/mL) was pipetted onto each hydrogel and incubated for 24 h at 37 °C and 5% CO_2_. The following day, the cells were fixed in 4% (*w*/*v*) paraformaldehyde (Sigma-Aldrich, Welwyn Garden City, UK) for 30 min and permeabilised in 0.5% (*v*/*v*) Triton X-100 solution (Sigma-Aldrich, Welwyn Garden City, UK) in PBS for 15 min. Cell morphology and cytoskeleton arrangement was assessed using Alexa Fluor™ 488 Phalloidin (Invitrogen™, A12379, Loughborough UK) as previously described [13]. RGB images were split in their channels and green channel images were used for morphological analysis by using ImageJ, version 1.51 [57]. In particular, images were threshold using the Huang’s algorithm, and touching cells were separated through a watershed algorithm. Cell adhesion and spreading were evaluated in terms of number of spread cells (%) and aspect ratio (major cell axis/minor cell axis).

### 4.10. Integrin-Dependent Cell Adhesion Assay

Cell adhesion on different modified hydrogels was also assessed in the presence of 10 μg/mL of the function-blocking monoclonal antibody mAb13 (Sigma-Aldrich, Welwyn Garden City, UK, MABT821) which inhibits the interaction between collagen and the β1-integrin subunit. Following the procedure described by Tuckwell [47], the antibody was diluted 10-fold in warm serum-free DMEM, and the cells were incubated for 30 min in the presence of antibody, before seeding. The effect of the inhibition on the cell spreading assay was evaluated as described in Section 4.9.

### 4.11. Statistical Analysis

All quantitative values are presented as mean ± standard deviation. All experiments were performed using at least three replicates. Data were plotted using Origin^®^ 2019b [58] and compared using an unpaired t test, unless stated otherwise. One level of significance was used: *p* < 0.05 (* or #, where appropriate).

## Figures and Tables

**Table 1:** Proteins and peptides used for hydrogel formation and modification. Sequences are shown with standard single amino acid symbols, plus O for 4-hydroxyproline. Capping groups: Fmoc, fluorenylmethoxycarbonyl protecting group; Ac, N-terminal acetylation; NH_2_, C-terminal amidation.

Molecule	Sequence/Access IDs (Integrin Binding Sites in Bold Type)	Amino Acids (aa)	*M_w_* (kDa)	Isoelectric Point (pI)
Fmoc-FF/S hydrogel Fmoc-FF peptide Fmoc-S peptide	Fmoc-FF Fmoc-S	2 1	0.53 0.33	7.81 7.81
Rat tail collagen ^1^ α_1_ (I) chain α_2_ (I) chain	P02454, NP_445756 P02466, NP_445808	1056 ^3^ 1040 ^3^	300 ^2^	9.52
GFOGER peptide	Ac-GPCGPPGPPGPPGPPGPPGFOGERGPPGPPGPPGPPGPPGPC-NH_2_	42	11.2 ^2^	6.96
DCol1 recombinant protein	MGSHHHHHHSGLVPRGSGPPGPPGPQGPAGPRGEPGPAGPKGEPGPAGPPGFPGERGPPGPQGPAGPIGPKGEPGPIGPQGPKGDPGETQIRFRLGPASIIETNSHGWFPGTDGALITGLTFLAPKDATRVQVFFQHLQVRFGDGPWQDVKGLDEVGSDTGRTGE	165	50.0 ^2^	6.97
eGFP recombinant protein	MGSSHHHHHHSSGLVPRGSHMVSKGEELFTGVVPILVELDGDVNGHKFSVSGEGEGDATYGKLTLKFICTTGKLPVPWPTLVTTLTYGVQCFSRYPDHMKQHDFFKSAMPEGYVQERTIFFKDDGNYKTRAEVKFEGDTLVNRIELKGIDFKEDGNILGHKLEYNYNSHNVYIMADKQKNGIKVNFKIRHNIEDGSVQLADHYQQNTPIGDGPVLLPDNHYLSTQSALSKDPNEKRDHMVLLEFVTAAGITLGMDELYK	259	29.1	6.61

^1^ Rat tail collagen is predominantly type I collagen, a heterotrimer made of two α_1_ (I) chains and one α_2_ (I) chain. ^2^ Molecular weights of the trimeric collagen molecules. ^3^ The amino acid number counts correspond to the processed, mature chains, of type I collagen.

## Supplementary Materials

The following supporting information can be downloaded at: https://www.mdpi.com/article/10.3390/gels8050254/s1, Figure S1: SDS-PAGE analysis of the proteins incorporated into the Fmoc-FF/S hydrogels, Figure S2: Rheological amplitude sweep test of Fmoc-FF/S (shear strain range of 0–100% 1 Hz) combined with different proteins, Figure S3: Optimisation of the concentration for mab13 anti-β1 subunit antibody.
