# Supplementary material for: Incorporation of Natural and Recombinant Collagen Proteins within Fmoc-Based Self-Assembling Peptide Hydrogels
Source: Gels. 2022 Apr 21;8(5):254. doi: 10.3390/gels8050254 (PMC9140497; doi:10.3390/gels8050254)
Supplement: Supplementary file 1 [file gels-08-00254-s001.zip › gels-1692037-supplementary.pdf]

gels

MDPI

Article

Incorporation of Natural and Recombinant Collagen Proteins within Fmoc-Based Self-Assembling Peptide Hydrogels

Mattia Vitale, Cosimo Ligorio, Ian P. Smith, Stephen M. Richardson, Judith A. Hoyland and Jordi Bella *

Supplementary Information

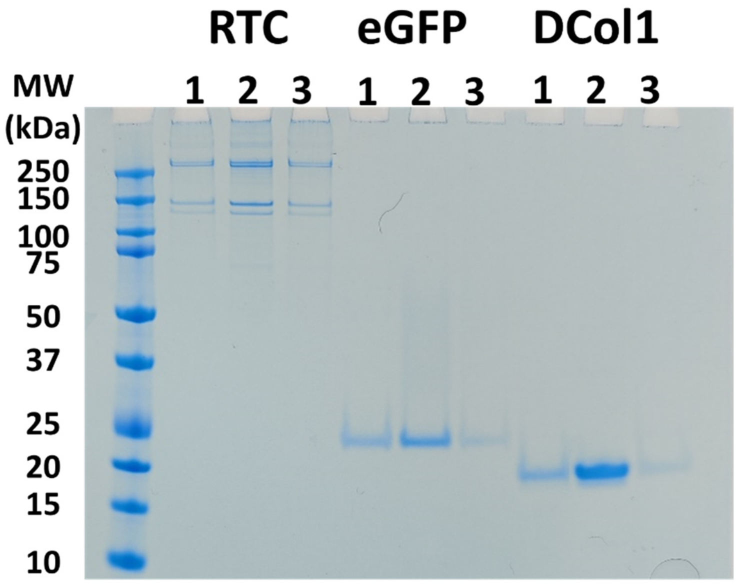

Figure S1. SDS-PAGE analysis of the proteins incorporated into the Fmoc-FF/S hydrogels. Lanes: M, molecular weight markers; 1, protein stock solution; 2, protein incorporated into the hydrogel; 3, remaining protein into the well.

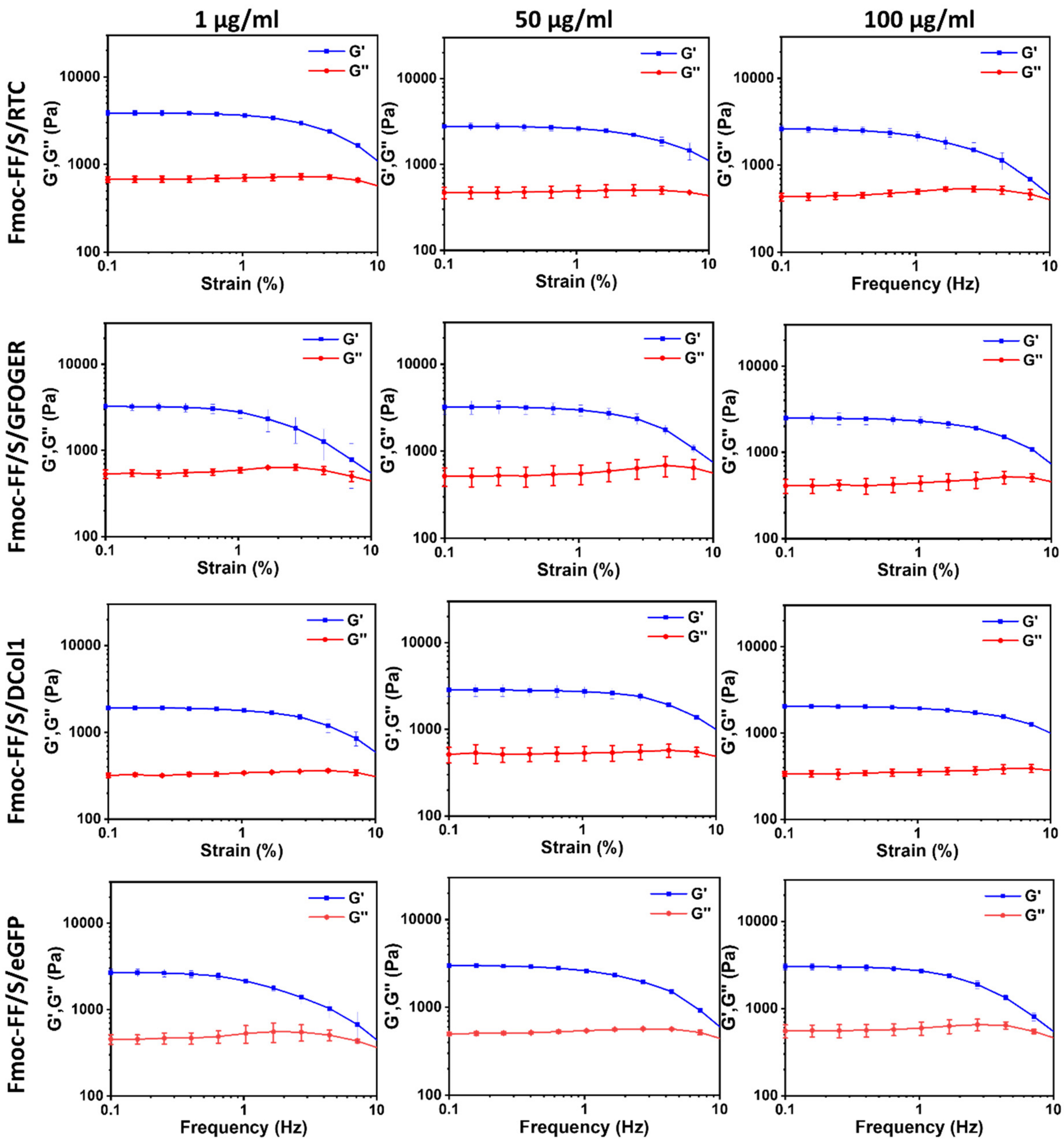

Figure S2. Rheological amplitude sweep test of Fmoc-FF/S (shear strain range of 0–100 % 1 Hz) combined with different proteins. Measurments were repeated for each hybrid hydrogels at three different concentrations (1, 50, 100 µg/ml). Line blue represents the storage modulus (*G′*) of each hydrogel whereas the red line illustrates the loss modulus (*G″*).

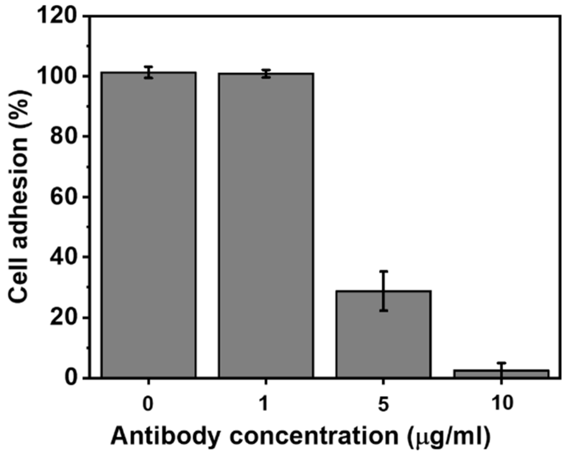

	Adhesion (%)	SD
**0 μg/ml**	101.2856515	1.848308537
**1 μg/ml**	100.879454	1.273858523
**5 μg/ml**	28.7263575	6.471772894
**10 μg/ml**	2.4936875	2.490480288

Figure S3. Optimisation of the concentration for mab13 anti-β1 subunit antibody. Briefly, 10 μg/ml of RTC (positive control) were used to coat the surface of a 24 multi-well plate. Protein excess was then removed with several washes in phosphate buffer, following blocking with 1% BSA (Bovine Serum Albumin) for 1h at RT. HT1080 were pre-incubated with different concentration of mab13 prior seeding. After 3 h cells were fixed in 4% PFA and cell adhesion was evaluated by manually counting.
